# A comparative study: Ultrasound-guided leverage reduction with internal fixation using Kirschner wires or elastic stable intramedullary nailing for severely displaced radial neck fractures in children

**DOI:** 10.1097/MD.0000000000035507

**Published:** 2023-10-27

**Authors:** Ji Wu, Xiantao Shen, Ping Zhang, Rui Zhou, Yanhong Li, Bingrong Tang, Xing Wu

**Affiliations:** a Department of Pediatric Orthopedics, Wuhan Children’s Hospital (Wuhan Maternal and Child Healthcare Hospital), Tongji Medical College, Huazhong University of Science & Technology, Wuhan, China; b Department of Medical Record Statistics, Wuhan Children’s Hospital (Wuhan Maternal and Child Healthcare Hospital), Tongji Medical College, Huazhong University of Science & Technology, Wuhan, China.

**Keywords:** children, internal fixation, radial neck fractures, ultrasonography

## Abstract

Treatment of radial neck fractures (RNFs) in children, particularly those with severe displacement or angulation, remains controversial, largely due to the challenge of achieving optimal reduction without resorting to open reduction. This study aimed to assess the outcomes of ultrasonography (US)-guided percutaneous leverage reduction coupled with US-guided fixation using either elastic stable intramedullary nail (ESIN) or Kirschner wire (KW) for severely displaced Judet type III and IV RNFs in children. We hypothesized that both strategies would be effective and aimed to identify the superior approach. A total of 38 pediatric patients presenting with Judet type III and IV RNFs resulting from falls were treated surgically between January 2020 and January 2022. The cohort comprised 15 boys and 23 girls, aged on average 7.6 ± 2.3 (range: 2.8–11.3 years). The fractures were classified as type III (n = 28) and type IV (n = 10). The patients were divided into 2 treatment groups: ESIN group (n = 15; treated with US-guided percutaneous leverage reduction and ESIN fixation) and the KW group (n = 23; treated with US-guided percutaneous leverage reduction and KW fixation). Variables such as surgical time, frequency of intraoperative radiography, fracture healing time, hospitalization costs, radiographic outcomes, and functional elbow scores were analyzed. Most fractures demonstrated both clinical and radiographic evidence of complete healing within 7 weeks. Based on the Tibone and Stoltz classification (Tibone J, Stoltz M. Fractures of the radial head and neck in children. J Bone Joint Surg Am. 1981;63:100–6), almost all patients had excellent or good clinical outcomes, with only one exception in the ESIN group. The KW group exhibited significantly lower hospitalization costs compared to the ESIN group [(9562.6 vs 12,043.6 + 7694.0)¥, *P* < .05]. Both groups required notably few intraoperative radiographic exposures (KW: 5.4 ± 2.1 times, ESIN: 4.0 ± 1.9 times, *P* < .05). No major complications were reported. However, one case of ESIN displacement and joint protrusion was noted. Our study suggests that US-guided percutaneous leverage reduction, combined with either ESIN or KW fixation, is an effective treatment for severely displaced radial neck fractures in children. Both treatment modalities resulted in notably few intraoperative radiographic exposures and yielded favorable clinical and radiological outcomes. The integration of US-guided leverage reduction and KW fixation is both cost-effective and safe.

## 1. Introduction

Fractures of the proximal radius constitute a mere 1% of all fractures and between 4.5 to 21% of pediatric elbow fractures, with radial neck fractures (RNFs) accounting for the majority of these cases.^[1]^ Although minimally displaced RNFs typically respond well to non-operative treatment, severely displaced or angulated fractures, such as Judet type III, IV, O’Brien type II, III, or AO type 21r–E/1.1 III,21r–E/2.1 III, 21r–M/3.1 III, often result in unsatisfactory outcomes when managed non-operatively.^[2–5]^ Most authors advocate for closed reduction for fractures with angulation >30°; however, achieving this reduction is challenging for severely angulated fractures. Open reduction permits anatomical reduction but involves a high risk of avascular necrosis of the radial head, and whenever possible closed reduction is preferred by the majority of surgeons.^[6,7]^ Moreover, after open reduction, the results are often cited as fair or poor.^[8]^ Percutaneous leverage reduction and internal fixation techniques have been further developed since their first introduction by Feray in 1969, offering a less invasive alternative.^[9]^ Métaizeau^[10]^ utilized intramedullary nailing for the reduction and fixation of radial neck fractures. While these minimally invasive procedures have been proved effective in many instances, challenges may still arise, particularly in cases of complete displacement. Guaranteeing anatomical or near-anatomical reduction is indeed challenging for completely displaced fractures. Ultrasound, increasingly used in pediatric musculoskeletal pathology evaluation, has demonstrated reliable diagnostic performance for long bone fractures in children when compared to conventional radiography.^[11,12]^ Additionally, several studies have confirmed the utility of ultrasound in assessing reduction adequacy.^[13,14]^ In literature, some studies^[15–17]^ have shown that ultrasound-guided diagnosis is reliable in detecting fractures of RNFs. Nevertheless, to our knowledge, we know of no previous studies reported in the literature comparing 2 different ultrasonography (US)-guided closed reduction and fixation methods in managing displaced radial neck fractures in children.

The aim of this study was to assess the outcomes of ultrasound-guided percutaneous leverage reduction and fixation using either elastic stable intramedullary nail (ESIN) or Kirschner wire (KW) for severely displaced Judet type III and IV radial neck fractures in children. The principal hypothesis of our study was that, in the surgical management of severely displaced radial neck fractures in children, US-guided percutaneous leverage reduction coupled with either ESIN or KW fixation is an effective treatment strategy. Simultaneously, by comparing 2 distinct methods of internal fixation, we also aimed to ascertain the superior therapeutic approach when coupled with ultrasonographic guidance.

## 2. Materials and Methods

### 2.1. Patient selection

The study was approved by our Institution’s Ethics Committee (2023R001-E01). The parents of the children authorized the use of their clinical data for this study. Thirty-eight children with RNFs operated at our hospital between January 2020 and 2022 were included in this retrospective study. Twenty-eight patients had Judet III fractures and 10 had Judet IV fractures. The inclusion criteria were as follows: (1) severely displaced RNFs (Judet III or Judet IV according to the Judet classification^[2]^) in children; (2) growth plates of the radial head are still open (<16 years); (3) follow-up for >1 year after surgery. The exclusion criteria were: (1) patients with a previous injury to the affected side or opposite elbow injury; (2) open fracture or pathological fracture.

### 2.2. Ultrasonic equipment

A GE LOGIOe ultrasonic instrument (GE Healthcare, Wauwatosa, WI) was employed for this study. The instrument utilized a 7.0–12.5 MHz variable frequency shallow surface probe of a flat type (GE Healthcare, Tokyo).

### 2.3. Surgical techniques

#### 2.3.1. US-guided percutaneous leverage reduction.

**Figure 1.**
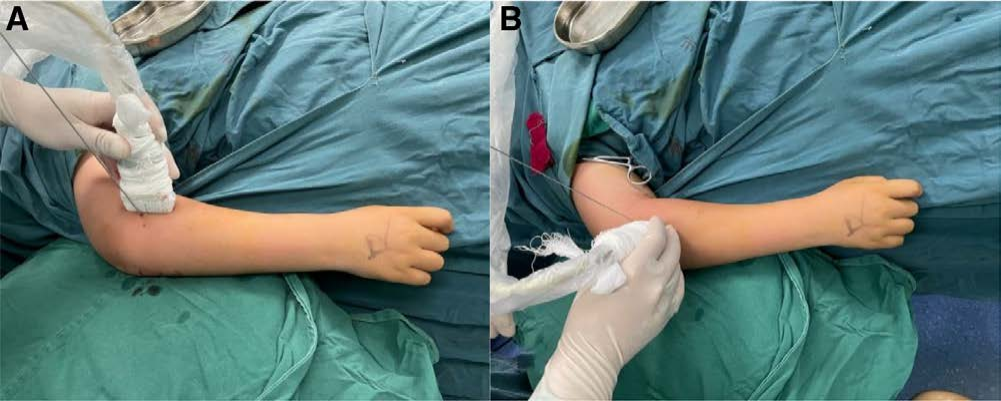
Patient and probe position of ultrasonography. (A and B) Photograph showing the patient and transducer positioning.

**Figure 2.**
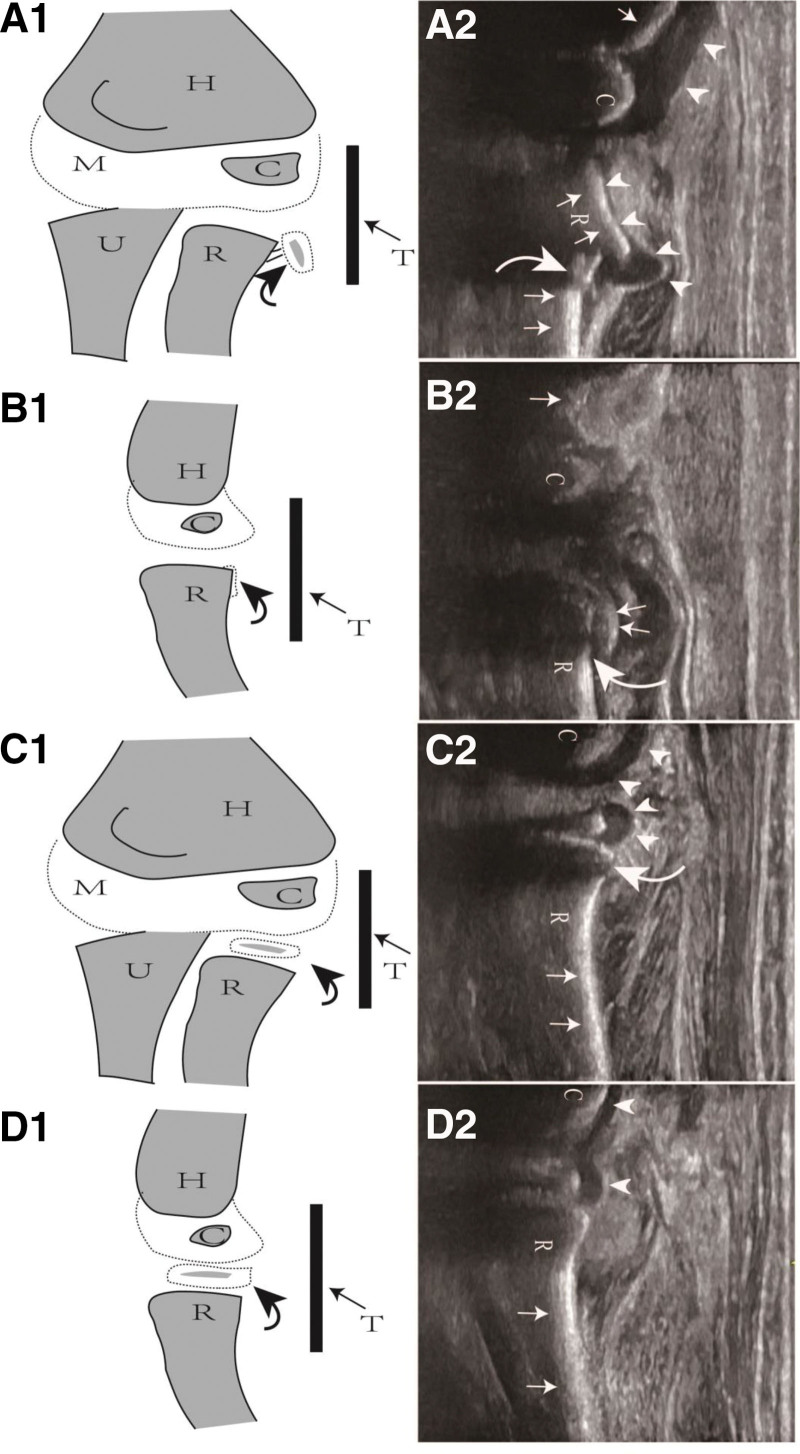
Longitudinal schematic drawing and ultrasound image depicting a Judet IV radial neck fracture. (A1) Prereduction coronal schematic drawing; (A2) prereduction coronal ultrasound image, indicating distinct angulation and displacement of the radial head towards the lower exterior; (B1) prereduction sagittal schematic drawing; (B2) prereduction sagittal ultrasound image, barely revealing the radial head; (C1) postreduction coronal schematic drawing; (C2) postreduction coronal ultrasound image, demonstrating the restored position of the radial head; (D1) postreduction sagittal schematic drawing; (D2) postreduction sagittal ultrasound image, similarly showing the restored position of the radial head. Arrowhead = cartilage surface, C = center of ossification of capitellum epiphysis, curved arrow = position of the radial neck fracture, H = humerus, M = medial, R = radius, straight arrow = cortex, T: transducer, U = ulna.

Under general anesthesia, patients were placed in a supine position with the injured forearm being pronated. A sufficient quantity of sterile coupling gel was applied to the ultrasonic probe, followed by wrapping the probe and wire tube with disposable sterile plastic sleeves. Sterile bandages were employed to secure part of the plastic sleeve on the probe surface (Fig. 1). Ultrasound facilitated the surgeons in accurately discerning the precise angulation of the radial neck fractures, allowing for continuous real-time monitoring of the reduction in multiple planes (Fig. 2). Depending on patient age and size, a 2.0-mm to 2.5-mm K-wire was introduced percutaneously under ultrasonographic guidance. Adapting the Kapandji technique,^[18]^ which is conventionally employed for distal radius fractures, the K-wire was inserted percutaneously from the distal and lateral aspect relative to the RNF site, leveraging it to achieve fracture reduction. The ultrasonic probe is predominantly oriented longitudinally, with the primary focus on examining the lateral and anterior aspects of the humeroradial joint to localize the sites of the radial neck fractures. Due to occasional posterior displacement of the radial head, the probe can be gently shifted posteriorly or subtly rotated based on real-time assessments, thereby obtaining the most definitive imaging of the radial head (Fig. 1). Ultrasound images could show the hypoechoic cartilage epiphysis of the proximal radius, the hyperechoic ossification center of the radial head, the hyperechoic bone cortex of the metaphysis, and the hyperechoic Kirschner wire (Fig. 2). After satisfactory reduction was confirmed on US, the assistant was instructed to maintain the leveraged Kirschner wire in position.

The choice of the surgical technique (percutaneous pinning or ESIN) depended on the surgeon’s abilities and preferences.

#### 2.3.2. US-guided fixation with K-wire.

**Figure 3.**
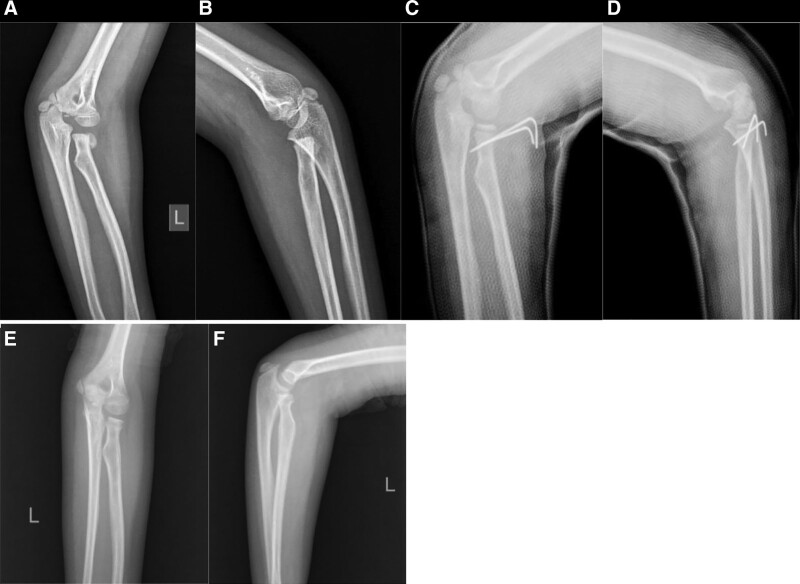
Representative case from the Kirschner Wire (KW) group: (A and B) initial anteroposterior (AP) and lateral radiographs demonstrating a Judet type IV radial neck fracture (RNF); (C and D) elbow radiographs taken on the first day postoperatively; (E and F) AP and lateral radiographs indicating complete fracture union following KW fixation.

Under the ultrasonic monitoring, an electric drill was utilized to drill a 1.5 mm diameter Kirschner wire through the lateral side of the fracture end to the opposite cortex for internal fixation. In 7 of the 23 cases, an additional K-wire was inserted in the same manner. The stability of the reduction and forearm rotation were assessed under ultrasonic guidance. Fluoroscopy was necessary to check the penetration into the far cortex. The K-wire was left protruding out of the skin and was bent over to prevent migration. A long-arm cast with the forearm in a neutral position was applied and maintained until K-wire removal (Fig. 3). The K-wire was removed along with the cast at 4 to 6 weeks after surgery in the outpatient clinic without sedation or anesthesia. After K-wire removal, an external fixed sling was applied for further 2 weeks in order to support the arm. Formal exercising began at least 6 weeks postsurgery, following the removal of the external fixed sling.

#### 2.3.3. US-guided fixation with ESIN.

The epiphyseal plate of distal radius was located by US, and a longitudinal 0.5 to 1.0 cm postero-radial skin incision was made proximally from the distal radial physis, carefully protecting the superficial branch of the radial nerve. Using a drill, an entry point was made 2 cm proximal to the physis. The diameter of the elastic stable intramedullary nails was selected according to child’s age, usually 1.5 to 2.0 mm. The nail was attached to a T-handle, and using C-arm fluoroscope, advanced proximally with gentle rotational movements until it reached the fracture. The reduction of the radial neck fracture was reconfirmed by US. If the proximal fragment match well, the nail was advanced with gentle taps across the fracture and up to the subchondral bone, avoiding penetration of the joint. If not, it could be percutaneously pried again under the guidance of US, or combined with Métaizeau technology,^[10]^ the T-handle could be rotated to fix the fracture of the radial neck until the reduction is satisfactory. Finally, the k-wire was pulled out, and the fracture was stabilized with one nail. The positions of fracture and internal fixation were then confirmed under fluoroscopy. A long-arm cast with the forearm in a neutral position was applied, and it was removed at 3 to 4 weeks after surgery. Rehabilitation was initiated after cast removal. The nail was extracted under general anesthesia in the inpatient department once satisfactory radiological healing was confirmed, typically between 3 to 6 months postoperatively.

### 2.4. Management of concurrent injuries

Plaster was applied for slightly displaced ulna fractures while Kirschner wires were utilized for severely displaced ulna or humerus fractures.

### 2.5. Evaluation tools

Information about sex, age, body mass index (BMI), fracture type, injured side, surgical time, hospitalization costs, healing time, and associated lesions were collected (Table 1). Angulation was measured as the angle between the fracture lines of the proximal and distal fragments. Displacement was also measured as the extent of lateral shift of the fragment by measuring the distance from the center of the radial head to a line along the axis of the upper radius.

**Table 1:** Patient’s demographics.

Variable	ESIN (n = 15)	KW (n = 23)	*P*
Sex (male:female)	6:9	9:14	>.99
Age (year)	7.2 ± 2.2	7.8 ± 2.3	.437
BMI (kg/m^2^)	14.5 ± 2.3	14.4 ± 2.3	.954
Fracture type (III/IV)			.473
Type III	10	18	
Type IV	5	5	
Follow-up (month)	16.2 ± 2.6	15.0 ± 2.7	.173
Accompanied injuries (with:without)	3:12	11:12	.101
Proximal ulnar fracture	1	10	
Ulnar shaft fracture	0	1	
Humeral medial condyle fracture	1	0	

ESIN = elastic stable intramedullary nail, KW = Kirschner wire.

Posttreatment clinical and radiographic data were collected during the follow-up period. Patients were followed up at the first month, second month, sixth month post-injury, and annually thereafter. Radiographically, reduction quality was assessed using the Métaizeau criteria.^[19]^ Clinically, the Tibone and Stoltz classification^[20]^was applied.

### 2.6. Statistical analysis

Comparative analysis was carried out between the 2 fixation groups. In the normal distribution (Shapiro–Wilk test), the Student *t* test was used for numerical data, and Mann–Whitney U test was used when they were not normally distributed. In the nonparametric distribution, we used the Mann–Whitney test and Spearman correlation. Categorical variables were evaluated with the chi-square (*χ*2) test or the Fisher exact test. All statistical analyses were performed using IBM SPSS Statistics for Windows (version 22.0; IBM, Armonk, NY). *P* < .05 was considered statistically significant.

## 3. Results

### 3.1. Patient characteristics

Our study comprised a total of 38 patients who met the stipulated inclusion and exclusion criteria. All patients had a follow-up duration exceeding 12 months, ranging from 12 months to 21.3 months. The average age of the patients was 7.6 ± 2.3 years (range: 2.8–11.3 years), with the mean age of ESIN-treated patients being 7.2 ± 2.2 years (range: 3.3–10.8 years) and for KW-treated patients, 7.8 ± 2.3 years (range: 2.8–11.3 years). Evaluating patient demographics, no significant differences were observed between groups in patient characteristics such as age, sex distribution, BMI, follow-up duration, fracture pattern, and associated injuries (Table 1).

### 3.2. Comparison of treatment outcomes: US-guided ESIN group vs US-guided KW group

The 38 patients who underwent treatment with the US-guided reduction and fixation methods were assessed functionally and radiologically (Table 2). At the last follow-up, based on the Tibone and Stoltz classification,^[20]^ the vast majority of patients generally expressed satisfaction with the long-term clinical outcomes. We observed excellent or good results in all patients, except one in the ESIN group (Table 2). The clinical outcomes for the KW group were excellent in 18 (78.3%) cases and good in 5 (21.7%) cases. For the ESIN group, the results were excellent in 11 (73.3%), good in 3 (20%), and fair in 1 (6%) case. No significant difference was found between the 2 groups (*P* = .656).

**Table 2:** Comparison of patient outcomes between groups.

Variable	ESIN (n = 15)	KW (n = 23)	*P*
Time of receiving surgery† (days)	4.0 ± 1.2	3.3 ± 1.5	.123
Surgical time‡ (minutes)	48.5 ± 31.2	48.2 ± 14.8	.509
Mean hospitalization costs§ (¥)	12,043.6 + 7694.0#	9562.6	<.001*
Times of X-radiographs‖	5.4 ± 2.1	4.0 ± 1.9	.001*
Reduction by Metaizeau’s grading			>.99
	11 excellent	17 excellent	
	4 good	6 good	
Time to union (weeks)	56 ± 10.9	42 ± 5.4	<.001*
Follow-up period (months)	16.3 ± 2.6	15.0 ± 2.7	0.173
Clinical outcomes by Tibone¶			0.656
	11 excellent	18 excellent	
	3 good	5 good	
	1 fair	0 fair	
Total complication rate, %	13%	8.70%	>.99
Fixation shift	1	0	
Malunion	1	0	
Stiffness	0	2	

ESIN = elastic stable intramedullary nail, KW = Kirschner wire.

† (Time of receive surgery): Time interval from injury to the start of surgical treatment.

‡ (Surgical time): Time spent on the initial surgical treatment.

§ (Hospitalization costs): All treatment costs for the first hospitalization (RMB).

‖ (Times of X-radiographs): Number of intraoperative radiographs.

¶ (Tibone): Tibone and Stoltz classification.

# Patients in the ESIN group underwent 2 surgeries, resulting in 2 hospital costs.

* Significant at *P* < .05.

**Figure 4.**
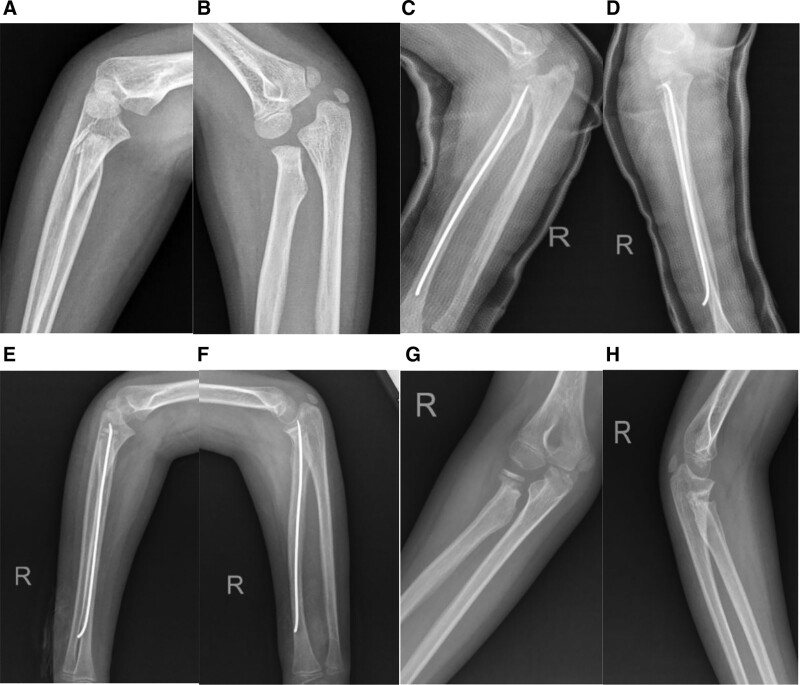
Case of ESIN shifting and joint protrusion: (A and B) initial AP and lateral radiographs demonstrating a Judet type IV radial neck fracture (RNF); (C and D) elbow radiographs taken on the first postoperative day; (E and F) AP and lateral radiographs at a 45-day postoperative follow-up, showing ESIN shifting and intrusion into the joint; (G and H) radiographs taken at a 6-month postoperative follow-up, revealing malunion of the radial neck.

Both treatment modalities implemented in our study demonstrated notably few intraoperative radiographic exposures, specifically (5.4 ± 2.1) times and (4.0 ± 1.9) times respectively (*P* < .05).In terms of radiological evaluations, the quality of reduction, as per the Métaizeau classification,^[19]^ was excellent in 11 (73.3%) patients and good in 4 (26.6%) of the ESIN group. In the KW group, excellent and good outcomes were noted in 17 (73.9%) and 6 (26.1%) of patients, respectively. These differences were not statistically significant (*P* > .999). Even though no statistically significant differences were found in postoperative complications like malunion and stiffness (Table 2), one case of ESIN shifting and joint protrusion was observed in the ESIN group during follow-up. The patient was a 7-year-old girl who displayed good alignment in her postoperative radiographs on the first postsurgery day. However, at the 45-day follow-up, ESIN shifting and intrusion into the joint were noticed (Fig. 4). She reported intraarticular pain and a limited range of motion in her elbow. Malunion, elbow pain, and dysfunction were noted after nail removal.

The average surgical time for the ESIN group was 48.5 ± 31.2 minutes compared to 48.2 ± 14.8 minutes for the KW group. This difference was not statistically significant (*P* = .509). There were no cases of compartment syndrome, vascular injury, treatment-related nerve injury, or pin infection. The K-wire group achieved significantly lower hospitalization costs [(9562.6 vs 12,043.6 + 7694.0)¥, *P* < .05]. In the ESIN group, a second surgery was required to remove the internal nail, resulting in additional hospitalization costs.

## 4. Discussion

The treatment of radial neck fractures in children remains controversial, particularly in cases with severe displacement or angulation. This study aimed to evaluate the long-term clinical and radiographic outcomes of RNF patients after receiving US-guided percutaneous leverage reduction and fixation. Overall, the majority of patients reported satisfaction with their long-term clinical results.

While several authors have reported positive results using the US technique,^[21–23]^ to our knowledge, no prior studies in existing literature have compared the clinical and radiographic outcomes, as well as complications, following US-guided intramedullary versus percutaneous pinning techniques. Concerning radiological evaluations, we assessed the quality of reduction using the Métaizeau classification.^[19]^ Our study’s results highlighted a high quality of reduction, with 28 cases rated as excellent and 10 cases rated as satisfactory, amounting to an excellent rate of 73.7% (28/38). The clinical outcomes, as assessed by the Tibone and Stoltz score,^[20]^ were also exceptional with 29 cases rated as excellent and 8 as satisfactory, culminating in an excellent rate of 76.3% (29/38). These findings are consistent with the work of other researchers. For instance, Daniele^[24]^ utilized traditional radiography-guided percutaneous k-wire leverage reduction and k-wire fixation for angulated radial neck fractures in children, reporting that 75% of patients exhibited excellent reduction based on Métaizeau’s score. Furthermore, excellent clinical results were achieved in 73.7% of patients in Daniele study, as evaluated by the Tibone and Stoltz score.^[20]^ Meanwhile, in our study, the use of ultrasound did not significantly lengthen the average surgical duration for either method, with the ESIN group recording 48.5 ± 31.2 minutes and the KW group, 48.2 ± 14.8 minutes, respectively (*P* = .509). Su et al^[25]^ reported a surgical duration of 64.5 ± 3.8 minutes for the treatment of pediatric radial neck fractures using leverage reduction and elastic intramedullary nailing under traditional fluoroscopy. Zhang^[26]^ adopted the traditional fluoroscopy-guided percutaneous k-wire leverage reduction technique in conjunction with elastic intramedullary nailing for the treatment of Judet type III and IV radial neck fractures, with an average operative time of 55.2 ± 11.2 minutes. None of the patients developed nonunion, avascular necrosis, infection, periarticular ossification, radioulnar synostosis, nerve injury, or premature physeal closure. The special complication was represented by the case of ESIN shifting and intruding into the joint at 45 days follow-up after the surgery, reported only in the ESIN group. This study represents the first to compare 2 different US-guided closed reduction and fixation methods for the treatment of displaced radial neck fractures in children.

The reduction of radiation dose in X-ray imaging has been recognized as a high priority in the medical community, especially for children who are the most vulnerable patient population. Our team has been dedicated to the minimally invasive treatment of pediatric fractures under ultrasound guidance for more than 9 years, and has achieved promising initial success, demonstrating the potential of this approach in improving clinical outcomes for young patients^.[27–32]^ Given ultrasound’s superior penetration through the epiphysis and the distinct grayscale difference between bone, cartilage, and soft tissue, it effectively displays the radial head, the proximal cortex of the radius, and the relationship between the radial head and the distal articular surface of the humerus. Normally, the epiphysis appears as a hypoechoic area (dark area), with the ossification center and bone cortex exhibiting hyperechoic areas (bright areas). The radiohumeral joint can be observed from anterior, posterior, and lateral planes. Under normal circumstances, regardless of direction, the bone cortex and epiphysis are continuous and smooth, transitioning directly or in an arc, with no step-off phenomenon. In children, the capitulum of the humerus and the radial head are unossified, displaying low-echo shadows similar to “double fists” in 3 longitudinal planes, also known as the “double breasts sign.” During adolescence, the notch of the superior articular fossa and the annular ligament of the radial head become more evident, exhibiting a characteristic “hat” sign. When a radial neck fracture occurs, a step-off phenomenon may arise due to interruption of the bone cortex or separation of the epiphysis. A distinct “crooked hat sign” can be seen from longitudinal examination (Fig. 2). Ultrasound images can directly display the fracture morphology, guiding the Kirschner wire to swiftly insert into the fracture’s broken end while effectively avoiding surrounding blood vessels, nerves, and other crucial tissues.

US holds several advantages over fluoroscopy in diagnosing and treating RNFs in children. First, US presents a technical advantage in identifying pediatric bones with incomplete ossification, especially the radial head in young children. Even with older children or teenagers, we can visualize the complete radial head using ultrasonography. At this juncture, ultrasonography can significantly aid in the reduction process. Several studies demonstrated high sensitivity (86.6–97%) and specificity (86.6–100%) for fractures of the forearm bone and humerus.^[33,34]^ Fractures can be sonographically identified alongside subperiosteal hematomas, deformities, cortical disruption, and reverberating echoes.^[35]^ In radial neck fractures, the reverberating echo of the radial head is clearly visible and allows for easy assessment of the angulation. US can also locate the epiphyseal plate of the distal radius, which is advantageous for determining the intramedullary needle entry point of the distal radius without radiation exposure. Second, we can identify the pin used for reduction and continuously monitor the process in multiple planes using US, which provides a clearer assurance of the reduction quality in radial neck fractures. For severely displaced and angulated radial neck fractures, achieving a satisfactory reduction solely using the Métaizeau technique^[10]^ can be challenging due to its practical technical limitations. However, under ultrasonographic guidance, the fracture can be real-time reduced by percutaneous leverage and swinging back and forth using a K-wire inserted into the proximal fragment. This ensures the proximal fragment matches well with the distal fragment and secures an anatomical or near-anatomical reduction. This dynamic monitoring mode reduces repeated leveraging and fluoroscopy, eliminates guesswork during prying, and consequently lessens iatrogenic injuries. While maintaining fracture reduction, internal fixation can be rapidly completed, shortening the operation time. Moreover, US lowers intraoperative radiation exposure for both children and surgeons. The ALARA (“as low as reasonably achievable”) radiation safety principle stresses that in all procedures performed on children, the radiation should be minimized or avoided whenever possible.^[36]^ Based on this principle, we apply US to the treatment of pediatric fractures to reduce the radiation exposure of children and surgeons.

We found that in comparison to conventional fluoroscopy-guided reduction methods, the ultrasound-guided percutaneous leverage reduction technique considerably decreased fluoroscopy exposure. A comparative example originates from a collaborative study conducted by 2 Italian medical institutions.^[24]^They employed a conventional fluoroscopy-guided percutaneous leverage reduction with Kirschner wire internal fixation to treat pediatric radial neck fractures. Continuous intraoperative fluoroscopy was necessary during the operation to guide the insertion and leverage of K-wires. This resulted in an average intraoperative fluoroscopy time of 32 seconds, with the longest fluoroscopy duration extending to 73 seconds. Another study^[25]^ used K-wires for percutaneous leverage reduction and ESIN internal fixation to treat radial neck fractures under the guidance of X-ray fluoroscopy, with an average fluoroscopy count of 28.3 ± 4.3 times. In stark contrast, our study saw both groups using ultrasound guidance, which brought down the average intraoperative fluoroscopy count to 5.4 ± 2.1 and 4.0 ± 1.9 times respectively. Therefore, the use of ultrasound guidance significantly reduced the need for fluoroscopy exposure. Our leverage reduction was carried out under real-time ultrasound guidance, and fluoroscopy was primarily used to assist in confirming fracture and internal fixation positions.

Before implementing this technique, it is essential for practitioners to undergo training in pediatric ultrasound imaging, specifically focusing on the morphology of the radial neck and the distal radius. By comparing ultrasound images of both the injured and uninjured sides, a more accurate diagnosis of fractures can be achieved. According to a study by Hedlin et al,^[37]^ physicians without prior ultrasound experience can achieve the same level of diagnostic ability as experienced ultrasound imaging physicians after attending a 1.5-hour course on ultrasound diagnosis of pediatric distal radius fractures.

Furthermore, a prospective observational study by Thomas et al^[38]^ demonstrated that, subsequent to a standardized formal training session of one hour, non-ultrasonographers were capable of executing ultrasound-guided detection of long-bone fractures with relative ease. It is worth noting that none of the physicians participating in the study had any previous experience in ultrasonography. After the training session, these physicians found it notably easy to identify mid-shaft fractures of the femur or humerus.

Despite the promising findings, the application of ultrasound in fracture treatment comes with its limitations, chiefly its inability to penetrate bone. This necessitates the use of fluoroscopy to confirm the location of internal fixation, given that ultrasound can only visualize the near cortex of the bone.

This study carries several limitations. Firstly, it is a retrospective study, thereby constrained by the nature of convenience sampling. Another potential limitation lies in the varying levels of training and scanning experience of the physicians conducting the preliminary scans, which could have affected the reliability of the outcome comparisons. While ultrasound-guided treatment appears promising in reducing radiation exposure, further prospective, randomized, and more extensive studies are needed to solidify these findings.

## 5. Conclusion

In conclusion, our study suggests that US-guided percutaneous leverage reduction coupled with either ESIN or KW fixation is an effective treatment strategy for managing severely displaced radial neck fractures in children. Both treatment modalities resulted in notably few intraoperative radiographic exposures and yielded favorable clinical and radiological outcomes. The integration of US-guided leverage reduction and KW fixation is both cost-effective and safe. Future studies with larger cohorts and longer follow-up periods may further validate these findings and possibly reveal additional advantages or limitations inherent to each treatment modality.

## Author contributions

**Conceptualization:** Bingrong Tang.

**Data curation:** Bingrong Tang.

**Formal analysis:** Bingrong Tang.

**Funding acquisition:** Xiantao Shen.

**Investigation:** Xiantao Shen, Rui Zhou, Yanhong Li.

**Project administration:** Xing Wu.

**Resources:** Xiantao Shen, Ping Zhang, Rui Zhou, Yanhong Li.

**Supervision:** Ping Zhang, Rui Zhou.

**Writing – original draft:** Ji Wu.
